# Supplementary material for: The global distribution and drivers of wood density and their impact on forest carbon stocks
Source: Nat Ecol Evol. 2024 Oct 15;8(12):2195–212. doi: 10.1038/s41559-024-02564-9 (PMC11618071; doi:10.1038/s41559-024-02564-9)
Supplement: Supplementary file 1 — Supplementary Figs. 1–11, Tables 1–5 and References. [file 41559_2024_2564_MOESM1_ESM.pdf]

nature ecology & evolution

Supplementary information https://doi.org/10.1038/s41559-024-02564-9

The global distribution and drivers of wood density and their impact on forest carbon stocks

In the format provided by the authors and unedited

Page	Supplementary Figures/Tables
1	**Supplementary Figure 1. Residual spatial autocorrelation (Moran's I) of the global wood density model along a distance gradient, assessed using generalized additive models.**
2	**Supplementary Figure 2. Performance and validation of the community-wide wood density (CWD) models.**
3	**Supplementary Figure 3. Uncertainty of wood density maps at different levels: all species (a), angiosperms-only (b), and gymnosperms-only (c).**
4	**Supplementary Figure 4. Representation of the training data for the wood density model considering all covariates.**
5	**Supplementary Figure 5. The partial effects of seven environmental covariates on CWD within tropical (a), temperate (b), boreal (c) and dryland (d) forests.**
6	**Supplementary Figure 6. Recursive partitioning trees for the univariate effects of human disturbance (a) and fire frequency (b) on CWD.**
7	**Supplementary Figure 7. Comparison of our wood density-based live tree biomass estimates with previous biomass estimations.**
8	**Supplementary Figure 8. Compatibility of species-level wood density estimates across nine databases.**
9	**Supplementary Figure 9. Comparison of our wood density map with the map from Yang et al.**
10	**Supplementary Figure 10. Variation coefficients of individual wood density measurements for each of the 5,527 species with three to ten observations.**
11	**Supplementary Figure 11. The relative relationship between the biome-level means of plot-level species numbers and total growing stock volume in each biome.**
12	**Supplementary Table 1. Biomass expansion factors (BEFs) for each biome based on the Eurasia Forest and BAAD database.**
13	**Supplementary Table 2. Forest type-level wood density statistics based on the community-wide wood density estimates generated in this study.**
14	**Supplementary Table 3. Biome-level wood density statistics based on the community-wide wood density estimates generated in this study.**
15	**Supplementary Table 4. List of the 49 covariates used in the models of wood density in global forest.**
17	**Supplementary Table 5. Effects of the main covariates based on partial regression.**
18	**References**

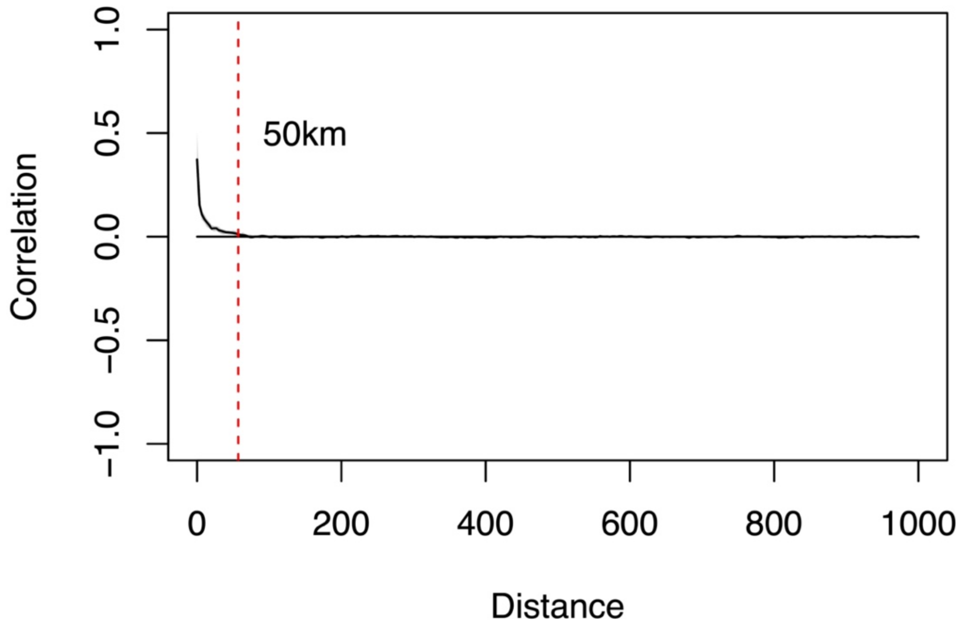

Supplementary Figure 1. Residual spatial autocorrelation (Moran's I) of the global wood density model along a distance gradient, assessed using generalized additive models. There was positive spatial autocorrelation up to a distance of ~50 km, and we applied this distance as buffer zone radius in the spatially-buffered zone-based bootstrap subsampling for modelling.

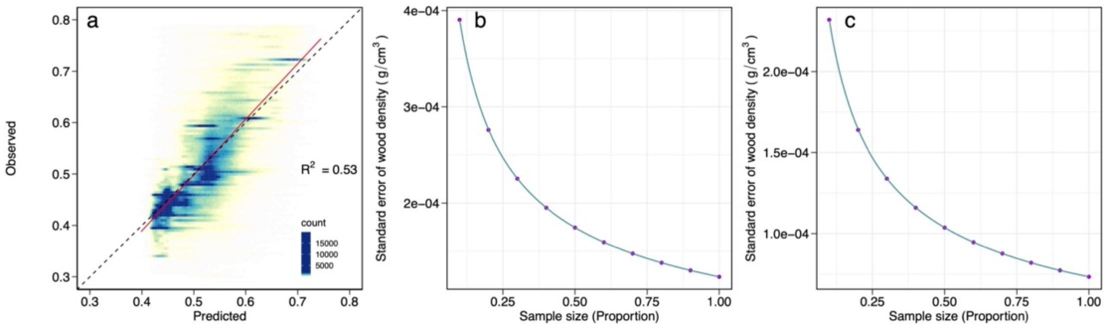

Supplementary Figure 2. Performance and validation of the community-wide wood density (CWD) models. a, The relationship between predicted and observed CWD values based on 10-fold cross validation for 200 spatially-buffered bootstrap models. The red line represents the linear regression, the dashed line represents the 1:1 line. **b, c**, Standard errors of the observed (b) and predicted (c) mean values of CWD in response to sample size. Each subsampling operation was repeated with 1000 random seeds for both the observed and predicted mean values, and the calculated standard errors of the mean are shown.

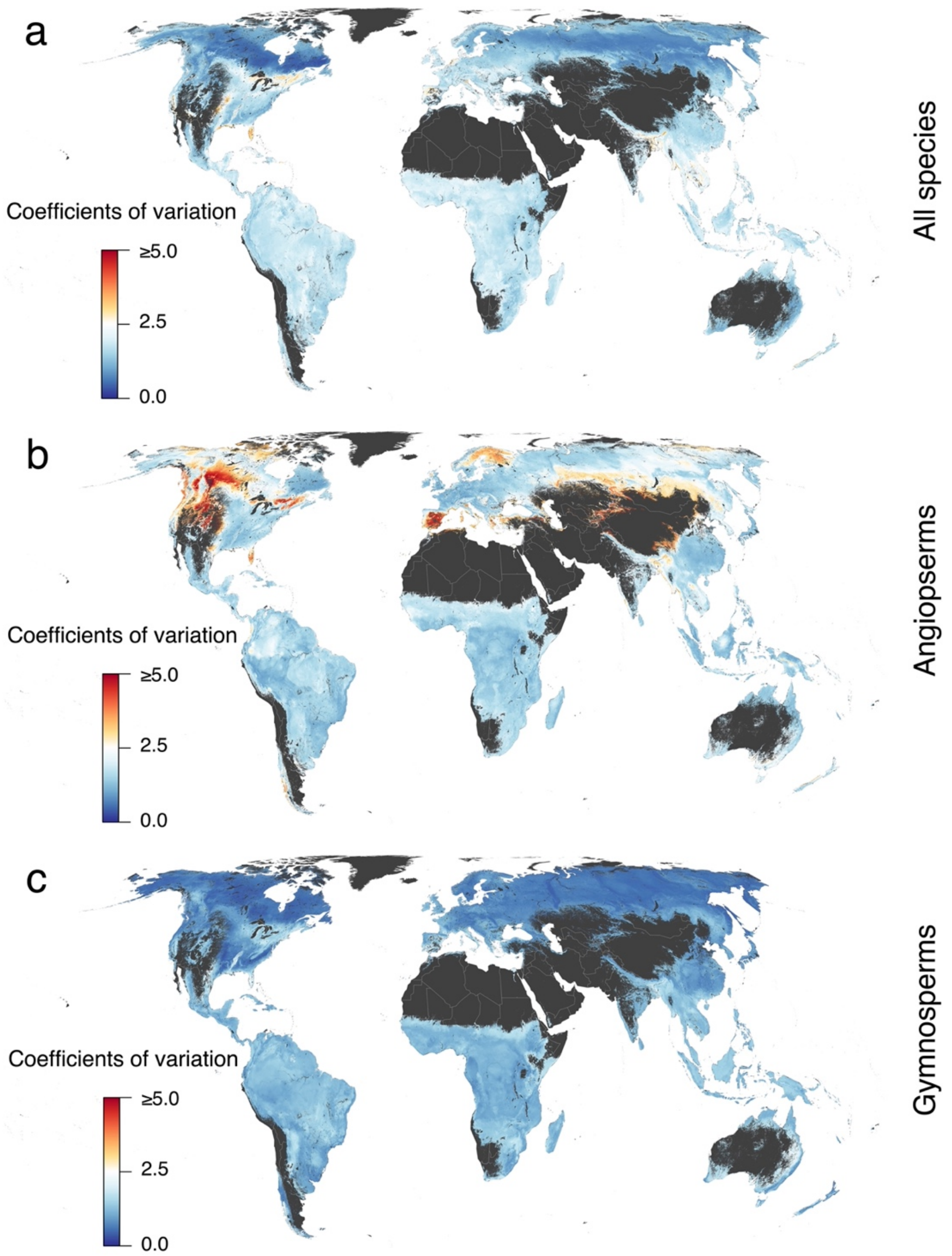

Supplementary Figure 3. Uncertainty of wood density maps at different levels: all species (a), angiosperms-only (b), and gymnosperms-only (c). Values represent bootstrapped coefficients of variation (standard deviation divided by mean) of CWD (a), angiosperm-only (b) and gymnosperms-only (c), respectively. Maps are projected at 30 arcsec (~1 km^2) resolution.

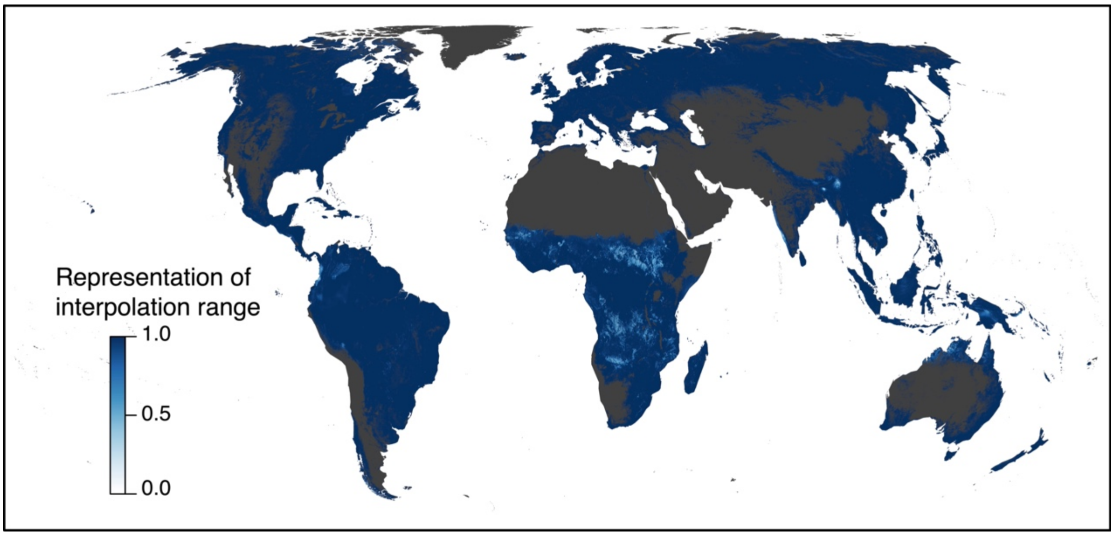

Supplementary Figure 4. Representation of the training data for the wood density model considering all covariates. To test for the extent of interpolation and extrapolation across all land pixels excluding Antarctica, we calculated the percentage of interpolation based on principal component analysis (PCA), that is, how often a pixel fell into the convex hull space of the bivariate combinations from the top principal components. **a**, To explore the representation of all covariates, we used 22 principal components with 231 combinations (see Methods). In total, 95.2% of the pixels fell within at least 95% of the PCA convex hulls.

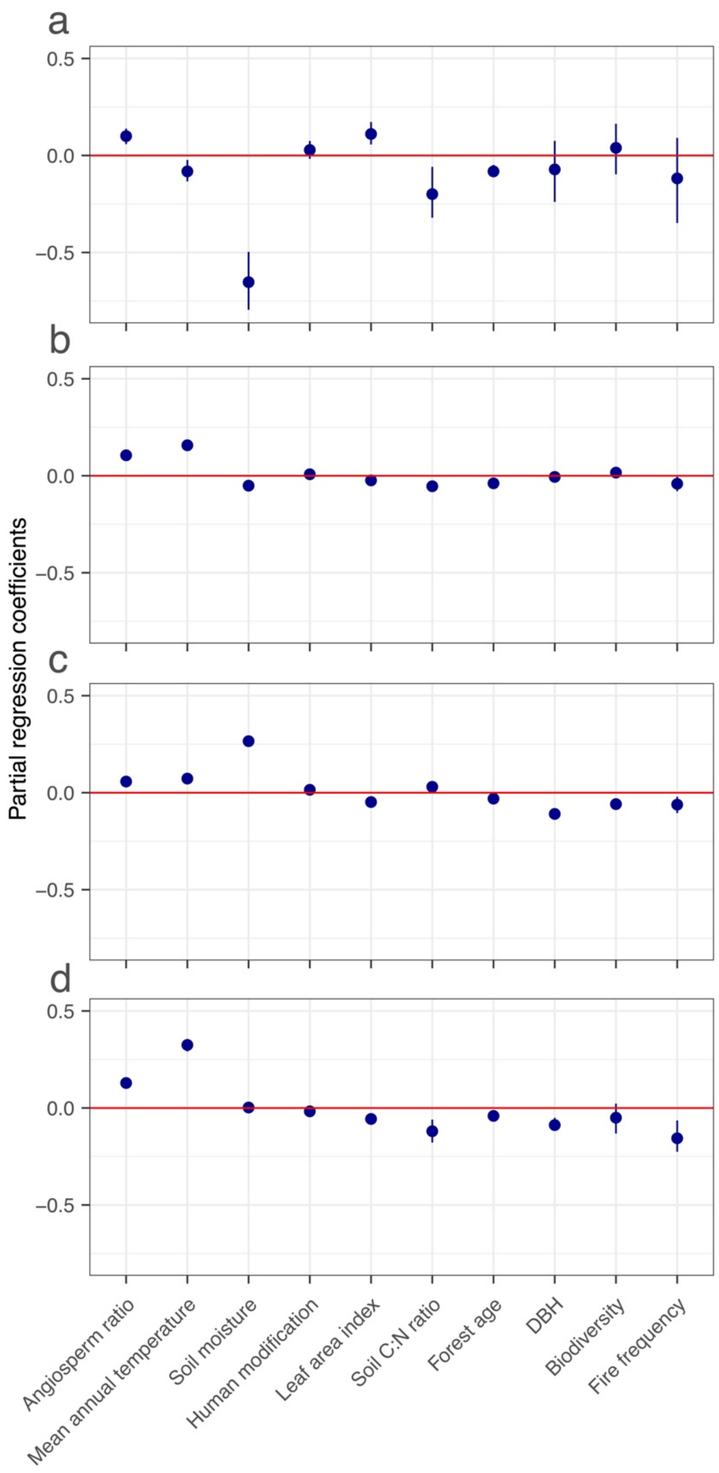

Supplementary Figure 5. The partial effects of seven environmental covariates on CWD within tropical (a), temperate (b), boreal (c) and dryland (d) forests. The red lines represent the partial regression coefficients ± 95% confidence intervals.

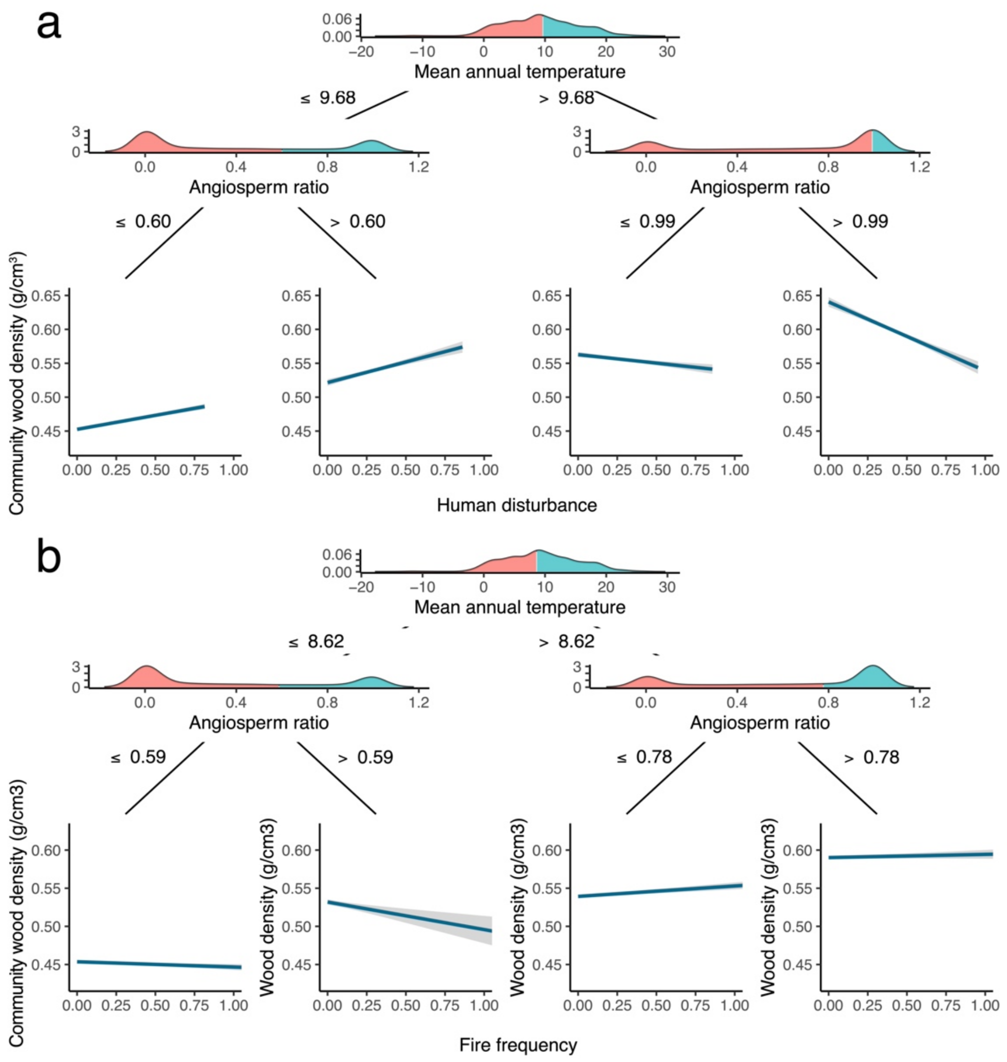

Supplementary Figure 6. Recursive partitioning trees for the univariate effects of human disturbance (a) and fire frequency (b) on CWD. The top four variables from a random forest model (Fig. 4a) were evaluated as potential split points. The number of independent observations contained in each terminal node was constrained to ≥2.5% of the total data (500 out of 19,000 observations) and the significance level was set to 0.01. Regression plots show slopes and 95% confidence intervals. The density plots at each splitting point illustrate the variable partitioning.

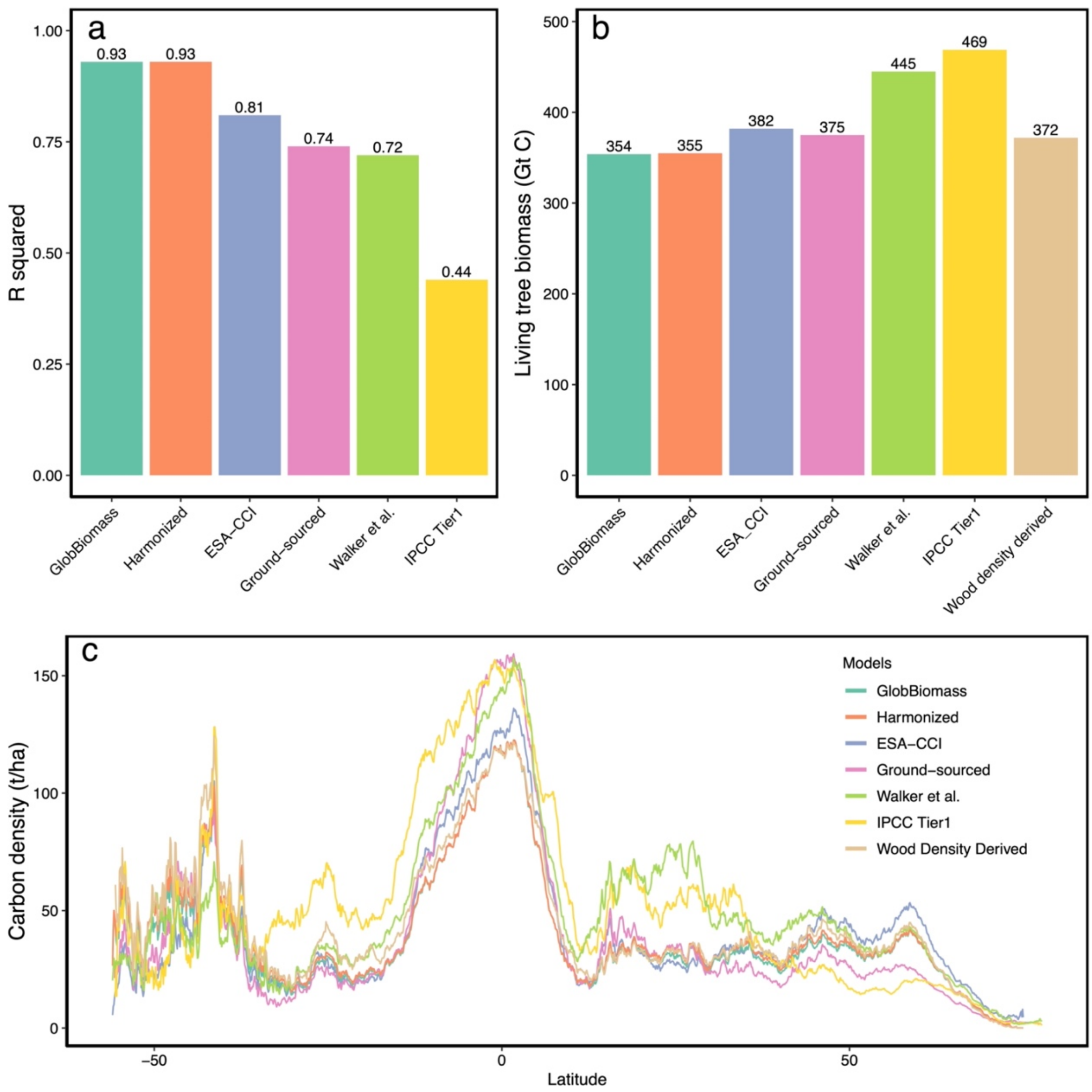

Supplementary Figure 7. Comparison of our wood density-based live tree biomass estimates with previous biomass estimations. a, R^2 values between previous biomass maps and our wood density-derived biomass map at 1 km^2 spatial resolution. The R^2s were calculated from 200,000 randomly sampled points. **b**, Total live biomass estimates from different biomass products. **c**. Comparison of live tree carbon along latitude between the seven biomass products. Lines were generated by calculating the mean of pixel-level (~1km^2) tree carbon densities for each 0.1 arc degree latitude.

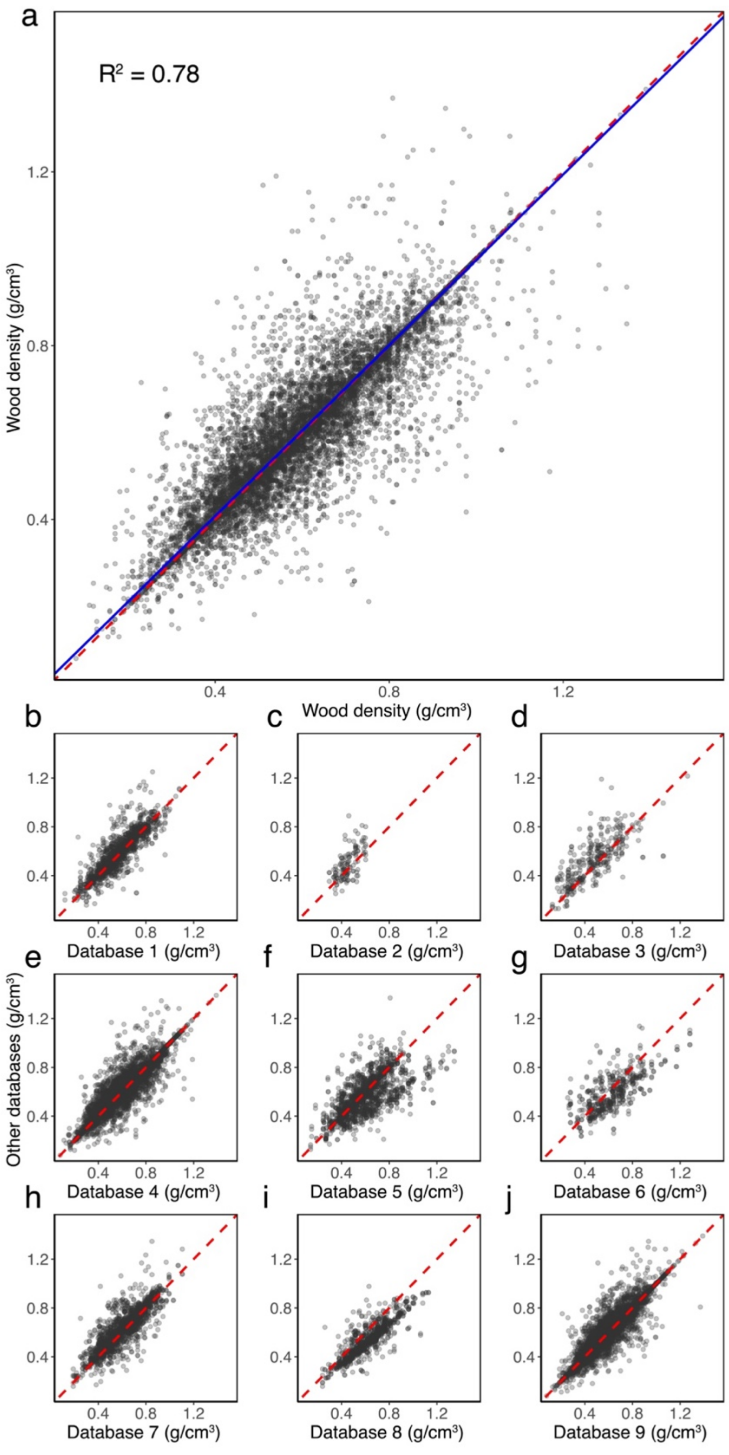

Supplementary Figure 8. Compatibility of species-level wood density estimates across nine databases. a. Distribution of species-level wood density pairs between any two of the nine databases. The blue line represents the regression line from reduced major axis regression ($R^2 = 0.78$); the red dashed line represents the identity line where y equals x. **b-j.** Distribution of species-level wood density pairs between one database and the other eight databases. Database 1 refers to ref[1], database 2 to ref[2], database 3 to ref [3], database 4 to refs[4,5], database 5 to published research articles (Supplementary 2), database 6 to refs[6,7], database 7 to ref[7], database 8 to ref[8], database 9 to ref[8].

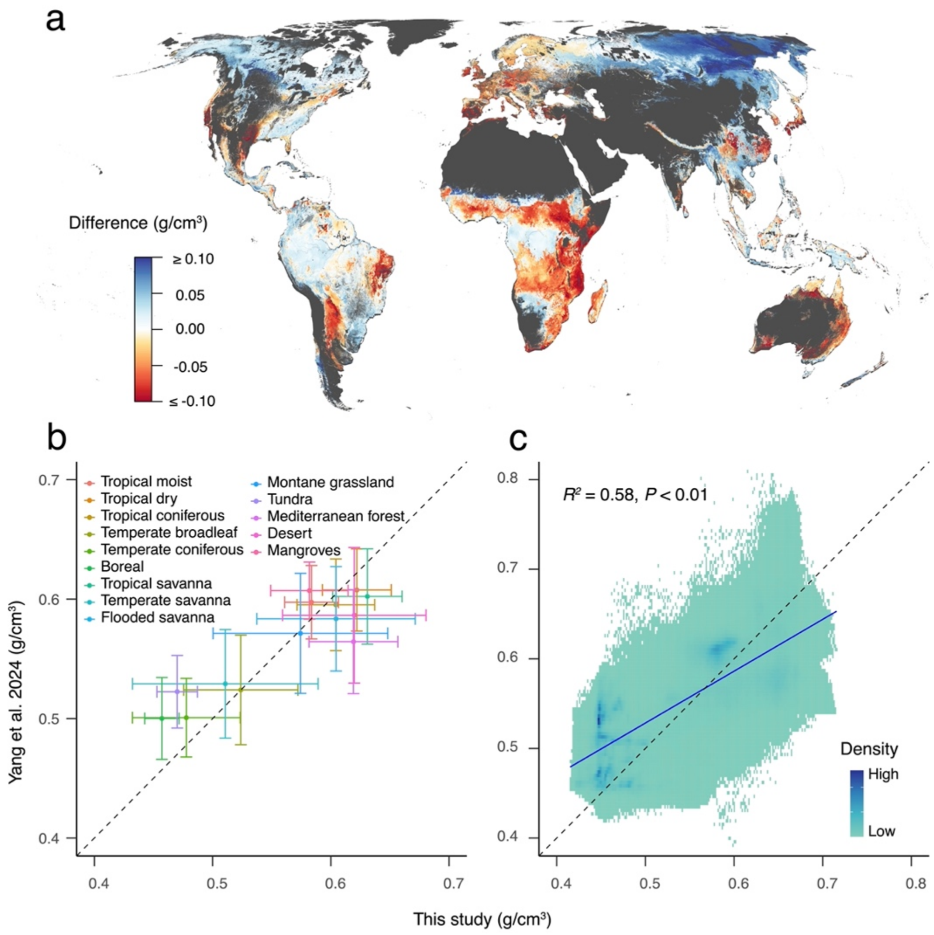

Supplementary Figure 9. Comparison of our wood density map with the map from Yang et al.[9]. a, Pixel-level comparison of wood density estimates at ~1km resolution. **b**, Comparison of the two wood density products at the biome level. The horizontal error bars represent the wood density range for each biome, calculated by adding and subtracting the standard deviation from the mean value of this study. Meanwhile, the vertical error bars indicate the wood density range for each biome, determined by adding and subtracting the standard deviation from the mean value reported by Yang et al.[9]. **C**, Scatterplot of the pixel-level pairwise comparisons (R^2= 0.58 and P<0.01, based on a linear regression model).

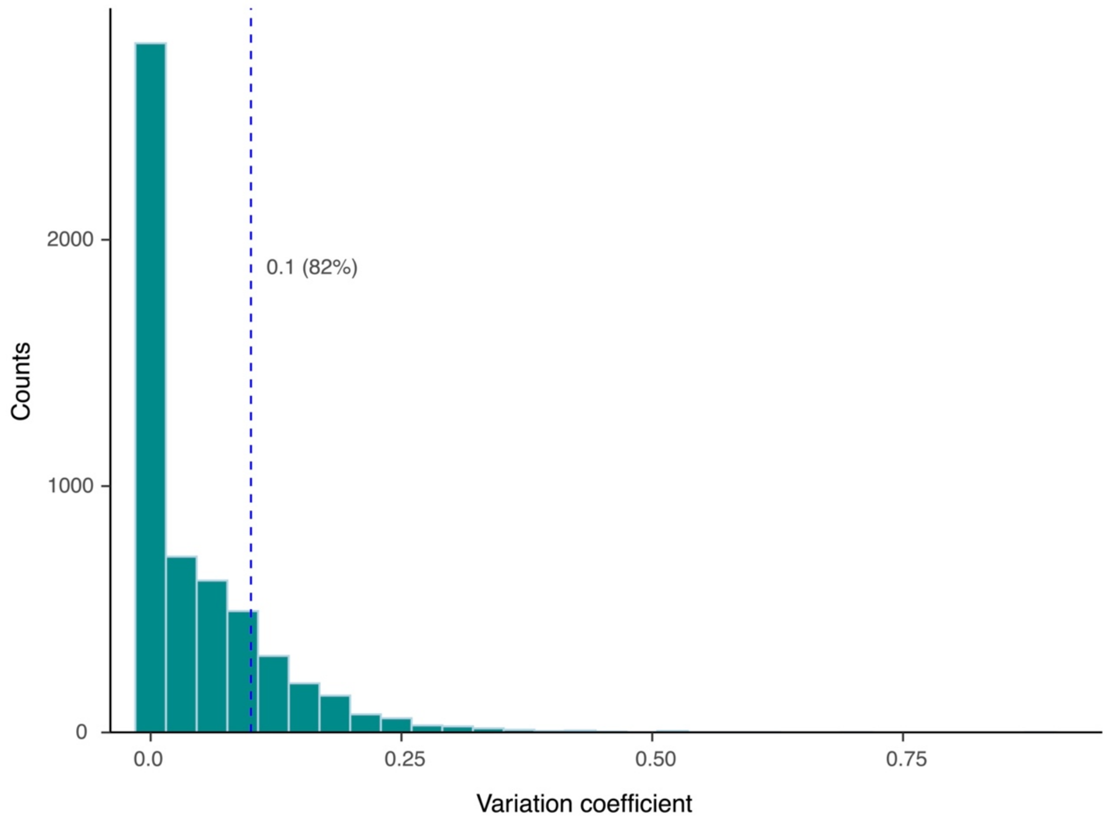

Supplementary Figure 10. Variation coefficients of individual wood density measurements for each of the 5,527 species with three to ten observations. The data are presented as frequency histogram, with the blue dashed line indicating a variation coefficient of 0.1. Overall, 82% of all species have variation coefficients below this threshold, and 48% of species have coefficients smaller than 0.01.

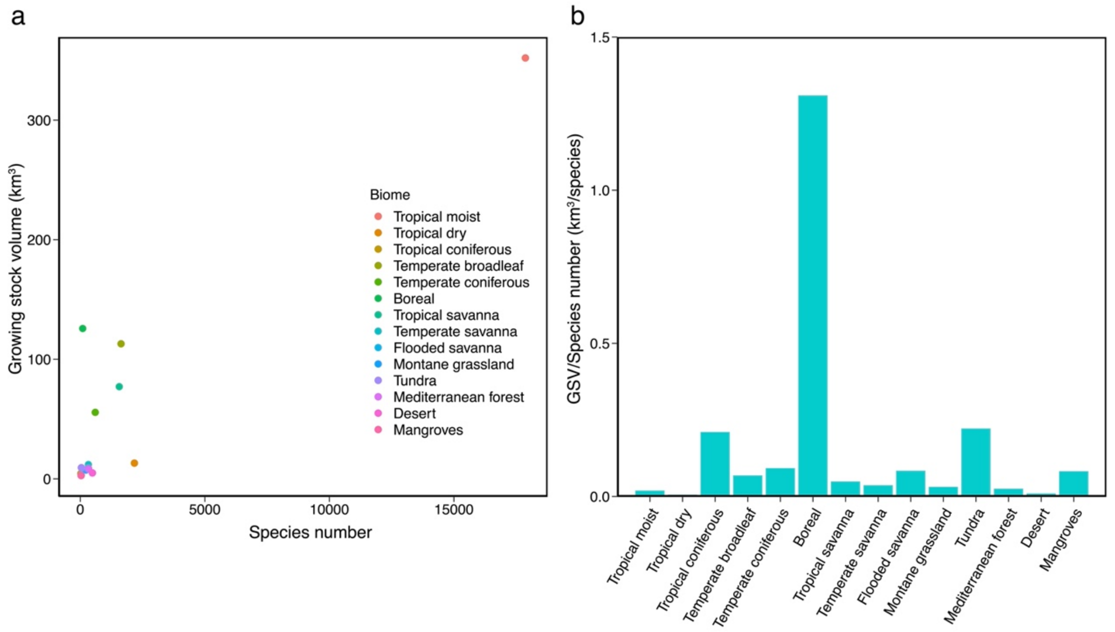

Supplementary Figure 11. The relative relationship between the biome-level means of plot-level species numbers and total growing stock volume in each biome. **A**, Scatterplot between species number and growing stock volume (km^3). **B**, Bar plot displaying the average growing stock volume per species in each biome. Growing stock volume data came from ref[10].

Supplementary Table 1. Biomass expansion factors (BEFs) for each biome based on the Eurasia Forest and BAAD database.

Biome	BEF	No. of records
Tropical & Subtropical Moist Broadleaf Forests	1.39	1180
Tropical & Subtropical Dry Broadleaf Forests	1.40	5
Tropical & Subtropical Coniferous Forests	1.33[a]	/
Temperate Broadleaf & Mixed Forests	1.35	4349
Temperate Conifer Forests	1.37	654
Boreal Forests/Taiga	1.33	1761
Tropical & Subtropical Grasslands, Savannas & Shrublands	2.25	7
Temperate Grasslands, Savannas & Shrublands	1.39	1173
Flooded Grasslands & Savannas	1.26	46
Montane Grasslands & Shrublands	1.32	140
Tundra	1.42	50
Mediterranean Forests, Woodlands & Scrub	1.32	41
Deserts & Xeric Shrublands	1.31	57
Mangroves	1.55[b]	6

[a]No information for tropical coniferous forests was available, and we therefore used the BEF from boreal forest.

[b]The BEF of mangroves was derived by averaging the BEF values from refs [11–14].

Supplementary Table 2. Forest type-level wood density statistics based on the community-wide wood density estimates generated in this study.

Forest type	Wood density (g/cm^3)
Tropical[1]	0.57 ± 0.10
Temperate[2]	0.52 ± 0.09
Boreal[3]	0.46 ± 0.05
Dryland[4]	0.59 ± 0.09

[1]Tropical including six biomes (tropical and subtropical moist broadleaf forest, tropical and subtropical dry broadleaf forest, tropical and subtropical coniferous forest, tropical and subtropical grassland, savanna and shrubland, flooded grassland and savanna, and mangroves).
[2]Temperate forest including four biomes (temperate broadleaf and mixed forest, conifer forest, temperate grassland, savanna and shrubland and montane grassland and shrubland).
[3]Boreal regions including two biomes (boreal forest/taiga and tundra).
[4]Dryland forest including two biomes (Mediterranean forest, woodland and scrub and desert and xeric shrubland).

Supplementary Table 3. Biome-level wood density statistics based on the community-wide wood density estimates generated in this study.

WWF Biome		Wood density (g/cm^3)
Simplified name	Full name	
Tropical moist	Tropical & Subtropical Moist Broadleaf Forests	0.58 ± 0.08
Tropical dry	Tropical & Subtropical Dry Broadleaf Forests	0.59 ± 0.10
Tropical coniferous	Tropical & Subtropical Coniferous Forests	0.60 ± 0.14
Temperate broadleaf	Temperate Broadleaf & Mixed Forests	0.53 ± 0.09
Temperate coniferous	Temperate Conifer Forests	0.49 ± 0.07
Boreal	Boreal Forests/Taiga	0.46 ± 0.05
Tropical savanna	Tropical & Subtropical Grasslands, Savannas & Shrublands	0.58 ± 0.11
Temperate savanna	Temperate Grasslands, Savannas & Shrublands	0.57 ± 0.10
Flooded savanna	Flooded Grasslands & Savannas	0.46 ± 0.08
Montane grassland	Montane Grasslands & Shrublands	0.57 ± 0.10
Tundra	Tundra	0.52 ± 0.06
Mediterranean forest	Mediterranean Forests, Woodlands & Scrub'	0.60 ± 0.09
Desert	Deserts & Xeric Shrublands	0.55 ± 0.08

Supplementary Table 4. List of the 49 covariates used in the models of wood density in global forest.

Variable	Type	Data resolution	Source and Reference
**Annual mean temperature**	Bioclimatic	~1km	CHELSA Ver 1.2 www.chelsa-climate.org Ref[15]
**Temperature annual range**	Bioclimatic	~1km	
**Temperature seasonality**	Bioclimatic	~1km	
**Isothermality**	Bioclimatic	~1km	
**Maximum temperature of warmest month**	Bioclimatic	~1km	
**Mean diurnal range**	Bioclimatic	~1km	
**Mean temperature of coldest quarter**	Bioclimatic	~1km	
**Mean temperature of driest quarter**	Bioclimatic	~1km	
**Mean temperature of warmest quarter**	Bioclimatic	~1km	
**Mean temperature of wettest quarter**	Bioclimatic	~1km	
**Minimum temperature of coldest month**	Bioclimatic	~1km	
**Precipitation seasonality**	Bioclimatic	~1km	
**Annual precipitation**	Bioclimatic	~1km	
**Precipitation of coldest quarter**	Bioclimatic	~1km	
**Precipitation of driest month**	Bioclimatic	~1km	
**Precipitation of driest quarter**	Bioclimatic	~1km	
**Precipitation of warmest quarter**	Bioclimatic	~1km	
**Precipitation of wettest month**	Bioclimatic	~1km	
**Precipitation of wettest quarter**	Bioclimatic	~1km	
**Solar radiation annual mean**	Climatic	~1km	WorldClim version 2. http://www.worldclim.com/version2 Ref[16]
**Windspeed annual mean**	Climatic	~1km	
**Water vapor Pressure annual mean**	Climatic	~1km	
**Inter-annual standard deviation of cloud cover**	Climatic	~1km	EarthEnv www.earthenv.org/cloud http://www.earthenv.org/topography Ref[17,18]
**Intra-annual SD of cloud cover**	Climatic	~1km	
**Annual mean of cloud cover**	Climatic	~1km	
**Eastness**	Topographic	~1km	
**Elevation**	Topographic	~1km	
**Northness**	Topographic	~1km	
**Profile curvature**	Topographic	~1km	
**Roughness**	Topographic	~1km	
**Slope**	Topographic	~1km	
**Aspect cosine**	Topographic	~1km	
**Topographic position index**	Topographic	~1km	
**Aspect sine**	Topographic	~1km	
**Aridity index**	Soil	~250m	Global Aridity Index and Potential Evapotranspiration (ET0) Climate Database version 2.0[19,20]
**Potential evapotranspiration (PET)**	Soil	~250m	

**Depth to water table**	Soil	~250m	SoilGrids https://soilgrids.org Ref[21]
**Absolute depth to bedrock**	Soil	~250m	
**Clay content 0-100cm**	Soil	~250m	
**Coarse fragments 0-100cm**	Soil	~250m	
**Sand content 0-100cm**	Soil	~250m	
**Cation exchange capacity**	Soil	~250m	
**Silt content 0-100cm**	Soil	~250m	
**Soil pH H_2O 0-100cm**	Soil	~250m	
**Soil nitrogen**	soil	~1km	
**C:N ratio**	soil	~1km	WISE30sec database [22]
**Soil moisture**	soil	~10km	GLDAS2.0[23]
**Cultivated and managed vegetation**	Anthropogenic	~1km	EarthEnv http://www.earthenv.org/landcover Ref[24]
**Urban builtup**	Anthropogenic	~1km	
**Human modification**	Anthropogenic	~1km	Kennedy et al.[25]
**Fire frequency**	Distrubance	~1km	MODIS Ref [26]
**Cropland**	Anthropogenic	~10km	HYDE 3.1 https://www.pbl.nl/en/image/links/hyde Ref [27,28]
**Grazing**	Anthropogenic	~10km	
**Pasture**	Anthropogenic	~10km	
**Rangeland**	Anthropogenic	~10km	
**Present tree cover**	Vegetative	~30m	Hansen et al., [29]
**NDVI**	Vegetative	~250	MODIS products Ref [30–32]
**EVI**	Vegetative	~500m	
**LAI**	Vegetative	~250m	
**FPAR**	Vegetative	~500m	
**NPP**	Vegetative	~1km	
**Tree density**	Vegetative	~1km	Crowther et al.[33]
**Canopy height**	Vegetative	~1km	Simard et al. [34]
**Forest age**	Vegetative	~1km	Besnard et al.[35]

Supplementary Table 5. Effects of the main covariates based on partial regression. This analysis examines the effect of each variable using the absolute values of the covariates. The 'Effects' column shows the corresponding change in community wood density for one unit change in the covariates.

Name of covariates	Unit of the change	Effects
Angiosperm ratio	10%	0.8%
Annual mean temperature	1 ℃	0.5%
Soil moisture	10 kg/m^2	-0.1%
C:N ratio	10%	-0.01%
Richness	10 species	0.006%
Lai	1 m^2/m^2	-0.04%
Human disturbance	10%	0.02%
Fire frequency	1 outbreak	-0.8%
Forest age	10 years	-0.1%

References

1. Brown, S. *Estimating Biomass and Biomass Change of Tropical Forests: A Primer*. vol. 134 (Food & Agriculture Org., 1997).
2. Schepaschenko, D. *et al.* A dataset of forest biomass structure for Eurasia. *Sci Data* **4**, 170070 (2017).
3. Falster, D. S. *et al.* BAAD: a Biomass And Allometry Database for woody plants. (2015).
4. Chave, J. *et al.* Towards a worldwide wood economics spectrum. *Ecol Lett* **12**, 351–366 (2009).
5. Zanne, A. E. *et al.* Data from: Towards a worldwide wood economics spectrum. *Ecology Letters* Preprint at https://doi.org/doi:10.5061/dryad.234 (2009).
6. Henry, M. *et al.* GlobAllomeTree: international platform for tree allometric equations to support volume, biomass and carbon assessment. *Iforest* **6**, 326–330 (2013).
7. Vieilledent, G. *et al.* New formula and conversion factor to compute basic wood density of tree species using a global wood technology database. *Am J Bot* **105**, 1653–1661 (2018).
8. Zhang, S.-B., Slik, J. W. F., Zhang, J.-L. & Cao, K.-F. Spatial patterns of wood traits in China are controlled by phylogeny and the environment. *Global Ecology and Biogeography* **20**, 241–250 (2011).
9. Yang, H. *et al.* Global patterns of tree wood density. *Glob Chang Biol* **30**, e17224 (2024).
10. Santoro, M. *et al.* GlobBiomass Global Above-Ground Biomass and Growing Stock Volume Datasets, available on-line at http://globbiomass.org/products/global-mapping. Preprint at (2018).
11. Ishil, T. & Tateda, Y. Leaf area index and biomass estimation for mangrove plantation in Thailand. in *IGARSS 2004. 2004 IEEE International Geoscience and Remote Sensing Symposium* vol. 4 2323–2326 (IEEE, 2004).
12. Lisboa, S. N., Guedes, B. S., Ribeiro, N. & Sitoe, A. Biomass allometric equation and expansion factor for a mountain moist evergreen forest in Mozambique. *Carbon Balance Manag* **13**, 1–16 (2018).
13. Njana, M. A. Indirect methods of tree biomass estimation and their uncertainties. *Southern Forests: a Journal of Forest Science* **79**, 41–49 (2017).
14. Magalhães, T. M. Live above-and belowground biomass of a Mozambican evergreen forest: a comparison of estimates based on regression equations and biomass expansion factors. *For Ecosyst* **2**, 1–12 (2015).
15. Karger, D. N. *et al.* Climatologies at high resolution for the earth's land surface areas. *Sci Data* **4**, 170122 (2017).
16. Fick, S. E. & Hijmans, R. J. WorldClim 2: new 1-km spatial resolution climate surfaces for global land areas. *International journal of climatology* **37**, 4302–4315 (2017).
17. Wilson, A. M. & Jetz, W. Remotely sensed high-resolution global cloud dynamics for predicting ecosystem and biodiversity distributions. *PLoS Biol* **14**, e1002415 (2016).
18. Amatulli, G. *et al.* A suite of global, cross-scale topographic variables for environmental and biodiversity modeling. *Sci Data* **5**, 180040 (2018).
19. Trabucco, A. & Zomer, R. J. Global Soil Water Balance Geospatial Database. CGIAR Consortium for Spatial Information. In, Published online, available from the CGIAR-CSI GeoPortal. *www. cgiar-csi. org* (2010).
20. Zomer, R. J., Trabucco, A., Bossio, D. A. & Verchot, L. V. Climate change mitigation: A spatial analysis of global land suitability for clean development mechanism afforestation and

reforestation. *Agric Ecosyst Environ* **126**, 67–80 (2008).
21. Shangguan, W., Hengl, T., de Jesus, J. M., Yuan, H. & Dai, Y. Mapping the global depth to bedrock for land surface modeling. *J Adv Model Earth Syst* **9**, 65–88 (2017).
22. Batjes, N. H. Harmonized soil property values for broad-scale modelling (WISE30sec) with estimates of global soil carbon stocks. *Geoderma* **269**, 61–68 (2016).
23. Rodell, M. *et al.* The global land data assimilation system. *Bull Am Meteorol Soc* **85**, 381–394 (2004).
24. Tuanmu, M.-N. & Jetz, W. A global, remote sensing-based characterization of terrestrial habitat heterogeneity for biodiversity and ecosystem modelling. *Global Ecology and Biogeography* **24**, 1329–1339 (2015).
25. Kennedy, C. M., Oakleaf, J. R., Theobald, D. M., Baruch-Mordo, S. & Kiesecker, J. Managing the middle: A shift in conservation priorities based on the global human modification gradient. *Glob Chang Biol* **25**, 811–826 (2019).
26. Giglio, L. MOD14A1 MODIS/Terra thermal anomalies/fire daily L3 global 1 km SIN grid V006. *NASA EOSDIS Land Processes DAAC, doi* **10**, (2015).
27. Klein Goldewijk, K., Beusen, A. & Janssen, P. Long-term dynamic modeling of global population and built-up area in a spatially explicit way: HYDE 3.1. *Holocene* **20**, 565–573 (2010).
28. Klein Goldewijk, K., Beusen, A., Van Drecht, G. & De Vos, M. The HYDE 3.1 spatially explicit database of human-induced global land-use change over the past 12,000 years. *Global Ecology and Biogeography* **20**, 73–86 (2011).
29. Hansen, M. C. *et al.* High-Resolution Global Maps of 21st-Century Forest Cover Change. *Science* vol. 342 850–853 (2013).
30. Didan, K., Munoz, A. B., Solano, R. & Huete, A. MODIS vegetation index user's guide (MOD13 series). *Vegetation Index and Phenology Lab, The University of Arizona* 1–38 (2015).
31. Myneni, R., Knyazikhin, Y. & Park, T. MOD15A2H MODIS/terra leaf area index/FPAR 8-day L4 global 500 m SIN grid V006. *NASA EOSDIS Land Processes DAAC* (2015).
32. Zhao, M., Running, S., Heinsch, F. A. & Nemani, R. MODIS-derived terrestrial primary production. in *Land remote sensing and global environmental change* 635–660 (Springer, 2010).
33. Crowther, T. W. *et al.* Mapping tree density at a global scale. *Nature* **525**, 201 (2015).
34. Simard, M., Pinto, N., Fisher, J. B. & Baccini, A. Mapping forest canopy height globally with spaceborne lidar. *J Geophys Res Biogeosci* **116**, (2011).
35. Besnard, S. *et al.* Mapping global forest age from forest inventories, biomass and climate data. *Earth Syst Sci Data* **13**, 4881–4896 (2021).
