# Supplementary material for: The global distribution and drivers of wood density and their impact on forest carbon stocks
Source: Nat Ecol Evol. 2024 Oct 15;8(12):2195–212. doi: 10.1038/s41559-024-02564-9 (PMC11618071; doi:10.1038/s41559-024-02564-9)
Supplement: Supplementary file 2 — Reporting Summary [file 41559_2024_2564_MOESM2_ESM.pdf]

nature portfolio

Corresponding author(s): Lidong Mo

Last updated by author(s): Jun 24, 2024

Reporting Summary

Nature Portfolio wishes to improve the reproducibility of the work that we publish. This form provides structure for consistency and transparency in reporting. For further information on Nature Portfolio policies, see our Editorial Policies and the Editorial Policy Checklist.

Statistics

For all statistical analyses, confirm that the following items are present in the figure legend, table legend, main text, or Methods section.

n/a	Confirmed	
☐	☒	The exact sample size (n) for each experimental group/condition, given as a discrete number and unit of measurement
☒	☐	A statement on whether measurements were taken from distinct samples or whether the same sample was measured repeatedly
☐	☒	The statistical test(s) used AND whether they are one- or two-sided<br>*Only common tests should be described solely by name; describe more complex techniques in the Methods section.*
☐	☒	A description of all covariates tested
☐	☒	A description of any assumptions or corrections, such as tests of normality and adjustment for multiple comparisons
☐	☒	A full description of the statistical parameters including central tendency (e.g. means) or other basic estimates (e.g. regression coefficient) AND variation (e.g. standard deviation) or associated estimates of uncertainty (e.g. confidence intervals)
☒	☐	For null hypothesis testing, the test statistic (e.g. F, t, r) with confidence intervals, effect sizes, degrees of freedom and P value noted<br>*Give P values as exact values whenever suitable.*
☒	☐	For Bayesian analysis, information on the choice of priors and Markov chain Monte Carlo settings
☒	☐	For hierarchical and complex designs, identification of the appropriate level for tests and full reporting of outcomes
☒	☐	Estimates of effect sizes (e.g. Cohen's d, Pearson's r), indicating how they were calculated

Our web collection on statistics for biologists contains articles on many of the points above.

Software and code

Policy information about availability of computer code

Data collection	Forest inventory plots data came from the Global Forest Biodiversity initiative (GFBi) database: https://www.gfbinitiative.org.<br>Wood density data came from Global Wood Density Database (Chave, J. et al. Towards a worldwide wood economics spectrum. Ecol. Lett. 12, 351–366 (2009).), TRY database (Kattge, J. et al. TRY plant trait database–enhanced coverage and open access. Glob. Chang. Biol. 26, 119–188 (2020)) and other sources(Schepaschenko, D. et al. A database of forest biomass structure for Eurasia. (2017); Falster, D. S. et al. BAAD: a Biomass And Allometry Database for woody plants. (2015); Henry, M. et al. GlobAllomeTree: international platform for tree allometric equations to support volume, biomass and carbon assessment. Iforest 6, 326–330 (2013); Vieilledent, G. et al. New formula and conversion factor to compute basic wood density of tree species using a global wood technology database. Am. J. Bot. 105, 1653–1661 (2018); Zhang, S.-B., Slik, J. W. F., Zhang, J.-L. & Cao, K.-F. Spatial patterns of wood traits in China are controlled by phylogeny and the environment. Glob. Ecol. Biogeogr. 20, 241–250 (2011)).
Data analysis	Used R and Google earth engine for data analysis. The corresponding references are listed below:<br>R Core Team (2023). _R: A Language and Environment for Statistical Computing_. R Foundation for Statistical Computing, Vienna, Austria. <https://www.R-project.org/>.<br>Gorelick, N., Hancher, M., Dixon, M., Ilyushchenko, S., Thau, D., & Moore, R. (2017). Google Earth Engine: Planetary-scale geospatial analysis for everyone. Remote Sensing of Environment.

For manuscripts utilizing custom algorithms or software that are central to the research but not yet described in published literature, software must be made available to editors and reviewers. We strongly encourage code deposition in a community repository (e.g. GitHub). See the Nature Portfolio guidelines for submitting code & software for further information.

Data

Policy information about availability of data

All manuscripts must include a data availability statement. This statement should provide the following information, where applicable:

- Accession codes, unique identifiers, or web links for publicly available datasets
- A description of any restrictions on data availability
- For clinical datasets or third party data, please ensure that the statement adheres to our policy

Data and code can be freely accessed from the GitHub link provided below, following the publication of the paper: https://github.com/LidongMo/GlobalWoodDensityProject.

Research involving human participants, their data, or biological material

Policy information about studies with human participants or human data. See also policy information about sex, gender (identity/presentation), and sexual orientation and race, ethnicity and racism.

Reporting on sex and gender	n/a
Reporting on race, ethnicity, or other socially relevant groupings	n/a
Population characteristics	n/a
Recruitment	n/a
Ethics oversight	n/a

Note that full information on the approval of the study protocol must also be provided in the manuscript.

Field-specific reporting

Please select the one below that is the best fit for your research. If you are not sure, read the appropriate sections before making your selection.

☐ Life sciences ☐ Behavioural & social sciences ☒ Ecological, evolutionary & environmental sciences

For a reference copy of the document with all sections, see nature.com/documents/nr-reporting-summary-flat.pdf

Ecological, evolutionary & environmental sciences study design

All studies must disclose on these points even when the disclosure is negative.

Study description	Here, we paired ~1.1 million ground-sourced forest inventory plots from the GFBi database with collated species-level wood density data to explore global variation in wood density among both angiosperm and gymnosperm trees. Using this large-scale observation approach, we tested competing hypotheses about the dominant factors driving wood density variation across global forests, including temperature, water availability, species composition and disturbances. This approach allowed us to test theoretical predictions of geographic variation and to create a global model of wood density. We calculated community-wide mean wood density by weighting the wood density of each individual observed in a forest plot by its basal area. To explore responses to anthropogenic and natural disturbance gradients, we integrated our observations with global information on human disturbance and fire frequency. Finally, we estimated the total live forest biomass by integrating our CWD map with spatially-explicit data on live tree volume, root mass fraction, and biome-level biomass expansion factors.
Research sample	Forest inventory plot data was downloaded from Global Forest Biodiversity initiative (GFBi) database: https://www.gfbinitiative.org Wood density data came from Global Wood Density Database (Chave, J. et al. Towards a worldwide wood economics spectrum. Ecol. Lett. 12, 351–366 (2009).), TRY database (Kattge, J. et al. TRY plant trait database–enhanced coverage and open access. Glob. Chang. Biol. 26, 119–188 (2020)) and other sources(Schepaschenko, D. et al. A database of forest biomass structure for Eurasia. (2017); Falster, D. S. et al. BAAD: a Biomass And Allometry Database for woody plants. (2015); Henry, M. et al. GlobAllomeTree: international platform for tree allometric equations to support volume, biomass and carbon assessment. Iforest 6, 326–330 (2013); Vieilledent, G. et al. New formula and conversion factor to compute basic wood density of tree species using a global wood technology database. Am. J. Bot. 105, 1653–1661 (2018); Zhang, S.-B., Slik, J. W. F., Zhang, J.-L. & Cao, K.-F. Spatial patterns of wood traits in China are controlled by phylogeny and the environment. Glob. Ecol. Biogeogr. 20, 241–250 (2011)).
Sampling strategy	n/a
Data collection	Forest inventory plot data was downloaded from Global Forest Biodiversity initiative (GFBi) database: https://www.gfbinitiative.org Wood density data came from Global Wood Density Database (Chave, J. et al. Towards a worldwide wood economics spectrum. Ecol. Lett. 12, 351–366 (2009).), TRY database (Kattge, J. et al. TRY plant trait database–enhanced coverage and open access. Glob. Chang.

	Biol. 26, 119–188 (2020)) and other sources(Schepaschenko, D. et al. A database of forest biomass structure for Eurasia. (2017); Falster, D. S. et al. BAAD: a Biomass And Allometry Database for woody plants. (2015); Henry, M. et al. GlobAllomeTree: international platform for tree allometric equations to support volume, biomass and carbon assessment. Iforest 6, 326–330 (2013); Vieilledent, G. et al. New formula and conversion factor to compute basic wood density of tree species using a global wood technology database. Am. J. Bot. 105, 1653–1661 (2018); Zhang, S.-B., Slik, J. W. F., Zhang, J.-L. & Cao, K.-F. Spatial patterns of wood traits in China are controlled by phylogeny and the environment. Glob. Ecol. Biogeogr. 20, 241–250 (2011)).
Timing and spatial scale	The estimates of wood density and environmental covariates are represented at approximately a 1km resolution.
Data exclusions	n/a
Reproducibility	Data and code can be freely accessed from the GitHub link provided below, following the publication of the paper: https://github.com/LidongMo/GlobalWoodDensityProject.
Randomization	n/a
Blinding	n/a

Did the study involve field work? ☐ Yes ☒ No

Reporting for specific materials, systems and methods

We require information from authors about some types of materials, experimental systems and methods used in many studies. Here, indicate whether each material, system or method listed is relevant to your study. If you are not sure if a list item applies to your research, read the appropriate section before selecting a response.

Materials & experimental systems

n/a	Involved in the study	
☒	☐	Antibodies
☒	☐	Eukaryotic cell lines
☒	☐	Palaeontology and archaeology
☒	☐	Animals and other organisms
☒	☐	Clinical data
☒	☐	Dual use research of concern
☐	☒	Plants

Methods

n/a	Involved in the study	
☒	☐	ChIP-seq
☒	☐	Flow cytometry
☒	☐	MRI-based neuroimaging

Dual use research of concern

Policy information about [dual use research of concern]

Hazards

Could the accidental, deliberate or reckless misuse of agents or technologies generated in the work, or the application of information presented in the manuscript, pose a threat to:

No	Yes	
☒	☐	Public health
☒	☐	National security
☒	☐	Crops and/or livestock
☒	☐	Ecosystems
☒	☐	Any other significant area

Experiments of concern

Does the work involve any of these experiments of concern:

No	Yes	
☒	☐	Demonstrate how to render a vaccine ineffective
☒	☐	Confer resistance to therapeutically useful antibiotics or antiviral agents
☒	☐	Enhance the virulence of a pathogen or render a nonpathogen virulent
☒	☐	Increase transmissibility of a pathogen
☒	☐	Alter the host range of a pathogen
☒	☐	Enable evasion of diagnostic/detection modalities
☒	☐	Enable the weaponization of a biological agent or toxin
☒	☐	Any other potentially harmful combination of experiments and agents

Plants

Seed stocks	n/a
Novel plant genotypes	n/a
Authentication	n/a
